# The Impact of Kidney Function on the Slow-Flow/No-Reflow Phenomenon in Patients Treated with Primary Percutaneous Coronary Intervention: Registry Analysis

**DOI:** 10.1155/2022/5815274

**Published:** 2022-11-30

**Authors:** Lidija Savic, Igor Mrdovic, Milika Asanin, Sanja Stankovic, Ratko Lasica, Gordana Krljanac, Dubravka Rajic, Damjan Simic

**Affiliations:** ^1^Faculty of Medicine, University of Belgrade, Belgrade, Serbia; ^2^Emergency Hospital & Cardiology Clinic, University Clinical Center of Serbia, Belgrade, Serbia; ^3^Center for Medical Biochemistry, Emergency Hospital, University Clinical Center of Serbia, Belgrade, Serbia

## Abstract

**Objective:**

The objective of this study is to analyze the impact of declining kidney function on the occurrence of the slow-flow/no-reflow phenomenon in patients with ST-elevation myocardial infarction (STEMI) treated with primary PCI (pPCI), as well as the analysis of the prognostic impact of the slow-flow/no-reflow phenomenon on short- and long-term mortality in these patients.

**Methods:**

We analyzed 3,115 consecutive patients. A value of the glomerular filtration rate (eGFR) at the time of admission of eGFR <90 ml/min/m^2^ was considered a low baseline eGFR. The follow-up period was 8 years.

**Results:**

The slow-flow/no-reflow phenomenon through the IRA was registered in 146 (4.7%) patients. Estimated GFR of <90 ml/min/m^2^ was an independent predictor for the occurrence of the slow-flow/no-reflow phenomenon (OR 2.91, 95% CI 1.25–3.95, *p* < 0.001), and the risk for the occurrence of the slow-flow/no-reflow phenomenon increased with the decline of the kidney function: eGFR 60–89 ml/min/m^2^: OR 1.94 (95% CI 1.22–3.07, *p* = 0.005), eGFR 45–59 ml/min/m^2^: OR 2.55 (95% CI 1.55–4.94, *p* < 0.001), eGFR 30–44 ml/min/m^2^: OR 2.77 (95% CI 1.43–5.25, *p* < 0.001), eGFR 15–29 ml/min/m^2^: OR 5.84 (95% CI 2.84–8.01, *p* < 0.001). The slow-flow/no-reflow phenomenon was a strong independent predictor of short- and long-term all-cause mortality: 30-day mortality (HR 2.62, 95% CI 1.78–3.57, *p* < 0.001) and 8-year mortality (HR 2.09, 95% CI 1.49–2.09, *p* < 0.001).

**Conclusion:**

Reduced baseline kidney function was an independent predictor for the occurrence of the slow-flow/no-reflow phenomenon, and its prognostic impact started with the mildest decrease in eGFR (below 90 ml/min/m^2^) and increased with its further decline. The slow-flow/no-reflow phenomenon was a strong independent predictor of mortality in the short- and long-term follow-up of the analyzed patients.

## 1. Introduction

Primary percutaneous coronary intervention (pPCI) is the gold standard of treatment in patients with acute ST-elevation myocardial infarction (STEMI), which significantly reduces short- and long-term mortality in such patients [1, 2]. Primary PCI restores normal flow (TIMI-3 flow) through the infarct-related artery (IRA) in around 90% of STEMI patients [1]. However, there is a small percentage of patients who continue to have impaired myocardial perfusion, despite the successful opening of the IRA [1, 3]. This phenomenon is called the slow-flow or no-reflow phenomenon [1]. The slow-flow/no-reflow phenomenon causes a larger necrosis zone in the myocardium, a greater risk for the occurrence of different complications, and a generally poorer prognosis in STEMI patients [1, 3–8]. The pathophysiology and the causes of the slow-flow/no-reflow phenomenon are complex and have not yet been fully explained [4, 7, 9–11].

Declining kidney function is a well-known predictor of both mortality and the occurrence of complications in STEMI patients treated with pPCI [12, 13]. This negative prognostic impact begins as soon as there is even the mildest decline in kidney function [14, 15]. There are studies that have proven kidney function to be an independent predictor for the occurrence of the slow-flow/no-reflow phenomenon [2, 5]. However, there are few studies analyzing the impact of different degrees of declining kidney function on the occurrence of this phenomenon.

The aim of this study is to analyze the prognostic impact of declining kidney function on the occurrence of the slow-flow/no-reflow phenomenon in patients with STEMI who had been treated with pPCI and to analyze the prognostic impact of the slow-flow/no-reflow phenomenon on short- and long-term all-cause mortality in these patients.

## 2. Method

### 2.1. Study Population, Data Collection, and Definitions

The present study enrolled 3,115 consecutive patients, hospitalized between February 2006 and January 2012, who were included in the prospective Clinical Center of Serbia STEMI Register. The purpose of the prospective Clinical Center of Serbia STEMI Register has been published elsewhere [16, 17]. The study protocol was approved by the local ethics committee (Ethics Committee of the University of Belgrade, Faculty of Medicine, Decision Number 470/II-4; Date: August 21, 2008). The study was conducted in accordance with the principles set forth in the Helsinki Declaration. The patients gave written informed consent, allowing their anonymized information to be published in this article.

In brief, the objective of the registry is to gather complete and representative data on the management and short- and long-term outcomes of patients with STEMI undergoing primary PCI in the center. All consecutive patients with STEMI, aged 18 or older, who were admitted to the Coronary Care Unit after undergoing pPCI in the Center were included in the register. For the purpose of this study, patients with cardiogenic shock at admission and patients on chronic hemodialysis were excluded.

Coronary angiography was performed via the femoral approach. Primary PCI and stenting of the infarct-related artery (IRA) were performed according to the standard technique. Aspirin, 300 mg, and clopidogrel, 600 mg, were administered to all eligible patients before pPCI. Selected patients with visible intracoronary thrombi were also given the GP IIb/IIIa receptor inhibitor during pPCI. The decision regarding the use of balloon predilatation or postdilatation, thrombus aspiration, as well as the type of stent was made at the physician's discretion. Flow grades were assessed according to TIMI (thrombolysis in myocardial infarction) criteria. The slow-flow/no-reflow phenomenon was categorized as postprocedural TIMI flow grades 0, 1, and 2 through the IRA, despite a residual stenosis of <50% and the absence of significant dissection, vasospasm, or a visible thrombus. After pPCI, patients were treated according to current guidelines.

Demographic, baseline clinical, angiographic, and procedural data were collected and analyzed. Hypertension, diabetes mellitus, and hyperlipidemia were recorded based on the patients' clinical information, their use of medication for these conditions, and their laboratory results. The creatinine level was measured in all patients immediately after admission, prior to primary PCI, i.e., prior to the application of iodine contrast. Kidney function was assessed at admission by estimating the glomerular filtration rate (eGFR) using the Modification of Diet in Renal Disease (MDRD) equation [18]. A value of <90 ml/min/m^2^ was considered as reduced baseline eGFR, and in relation to the eGFR value, reduced kidney function was classified into the following stages: mildly reduced (eGFR 60–89 ml/min/m^2^), mildly to moderately reduced (eGFR 45–59 ml/min/m^2^), moderately to severely reduced (eGFR 30–44 ml/min/m^2^), and severely reduced (eGFR 15–29 ml/min/m^2^) [17, 19].

An echocardiographic examination was performed within the first three days after pPCI. The left ventricular ejection fraction (LVEF) was assessed according to the biplane Simpson method in classical two- and four-chamber apical projections. LVEF was missing in 10% of patients. The missing data were imputed via the single imputation method.

Patients were followed-up at eight years after enrollment. Follow-up data were obtained through scheduled telephone interviews and outpatient visits. We analyzed all-cause mortality.

### 2.2. Statistical Analysis

Categorical variables were expressed as frequency and percentage, while continuous variables were expressed as the median value (med) with the 25^th^ and 75^th^ quartiles (IQR). An analysis of the normality of the data was performed using the Kolmogorov–Smirnov test. Baseline differences between the groups were analyzed using the Mann–Whitney test for continuous variables and the Pearson *X*^2^ test for categorical variables. Multiple logistic regression was used to define independent predictors of the slow-flow/no-reflow phenomenon (backward method, with *p* < 0.10 for entrance into the model). The Kaplan–Meier method was used for constructing the probability curves for eight-year survival, while the difference between patients with a postprocedural TIMI-3 flow and patients with the slow-flow/no-reflow phenomenon was tested with the logrank test. Multiple Cox regression (backward method, with a *p* < 0.10 for entrance into the model) was used to test the impact of the slow-flow/no-reflow phenomenon on short- and long-term mortality in the patients analyzed. A *p* value of <0.05 was considered significant. The SPSS Version 19 statistical software was applied (SPSS Inc., Chicago, IL).

## 3. Results

The slow-flow/no-reflow phenomenon through the IRA was registered in 146 (4.7%) patients. Baseline demographic, clinical, and laboratory data, angiographic and procedural characteristics, baseline kidney function, as well as the ejection fraction (EF) in patients with postprocedural TIMI-3 flow and patients with the slow-flow/no-reflow phenomenon, are shown in Table 1.

In comparison with patients who had a TIMI-3 flow, patients with the slow-flow/no-reflow phenomenon were older. The following characteristics were more frequently present in patients with the slow-flow/no-reflow phenomenon: previous coronary disease, diabetes, a longer duration of pain before first medical contact, heart failure at admission, atrial fibrillation, and complete atrioventricular block at admission, a lower value of systolic blood pressure and a higher heart rate at admission, three-vessel coronary disease, stenosis of the left-main coronary artery, and thrombotic occlusion of the IRA (preprocedural TIMI flow = 0) at the initial angiogram, and a lower value of EF. Patients with the slow-flow/no-reflow phenomenon had a lower average value of eGFR at admission as compared to patients with a TIMI-3 flow.

Data on therapy during hospitalization and at discharge are present in Table 2.

Predictors for the occurrence of the slow-flow/no-reflow phenomenon are present in Table 3.

After adjustment for variables defined in the univariate analysis as predictors of mortality, in the multiple logistic regression analysis, all stages of reduced kidney function were significantly associated with the slow-flow/no-reflow phenomenon. The risk of the occurrence of the slow-flow/no-reflow phenomenon increased with the decline of kidney function.

In-hospital, 30-day, one-year, and eight-year mortality were significantly higher in patients with the slow-flow/no-reflow phenomenon, as compared to patients with a TIMI-3 flow: in-hospital mortality of 30.1% vs. 2.9%, respectively, *p* < 0.001; 30-day mortality of 31.5% vs. 3.2%, respectively, *p* < 0.001; and eight-year mortality of 38.1% vs. 6.9%, respectively, *p* < 0.001.

**Figure 1:**
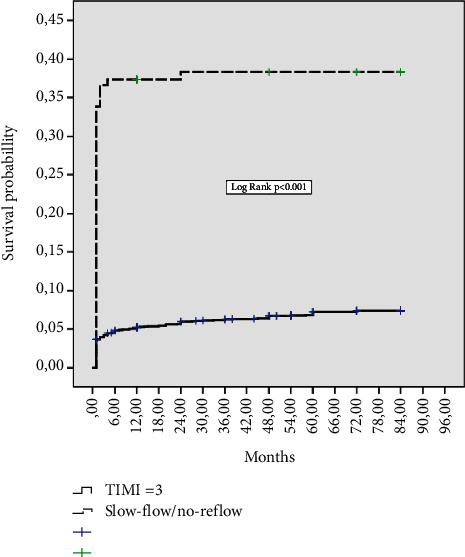
Kaplan–Meier curves showing mortality during an eight-year follow-up, according to the slow-flow/no-reflow phenomenon.

Kaplan–Meier curves estimating the probability of mortality during follow-up in patients with the slow-flow/no-reflow phenomenon and those with a TIMI-3 flow are shown in Figure 1.

The slow-flow/no-reflow phenomenon was a strong independent predictor of short-term, 30-day all-cause mortality (HR 2.62, 95% CI 1.78–3.57, *p* < 0.001) and long-term, one-year and eight-year all-cause mortality, as shown in Table 4.

## 4. Discussion

The results of the present study show that declining kidney function at admission was more frequently present in patients with the slow-flow/no-reflow phenomenon as compared to patients with a postprocedural TIMI-3 flow through the IRA. Decreased kidney function at admission was an independent predictor of the slow/flow-no-reflow phenomenon, and this independent impact increased with the decline of the kidney function, starting with the eGFR value of 90 ml/min/m^2^. The slow-flow/no-reflow phenomenon was a strong independent predictor for 30-day and eight-year all-cause mortality in the analyzed patients.

The incidence and clinical characteristics of patients with the slow-flow/no-reflow phenomenon in our study are in keeping with the data found in the literature, where the reported incidence of the slow-flow/no-reflow phenomenon ranges from 2.3% to as high as 30% [1–7, 11, 20]. The differences in the percentage of STEMI patients with the slow-flow or no-reflow phenomenon through the IRA are probably the result of different inclusion criteria, the characteristics of the patients themselves, the applied concomitant therapy, etc. [6]. In a study by Kurtul et al. analyzing the prognostic impact of mild and moderate renal impairment on the occurrence of the slow-flow/no-reflow phenomenon in patients with STEMI, the incidence of the slow-flow/no-reflow phenomenon was 17.2% [4]. The authors showed that the incidence of the slow-flow/no-reflow phenomenon increased with the decline in kidney function, while the eGFR (shown as a numerical variable) was an independent predictor of this phenomenon. As opposed to our study, patients with severely reduced kidney function (eGFR 15–30 ml/min/m^2^) were not analyzed, and the prognostic impact of different levels of reduced kidney function was not presented, while, through the application of the receiver operating characteristics (ROC) curve, it was found that the cut-off value of eGFR for the prediction of the slow-flow/no-reflow phenomenon was 64.9 ml/min/1.73 m^2^ [4]. In our study, we have shown that the independent prognostic impact of kidney function already begins with a mild level of kidney dysfunction, i.e., with an eGFR of <90 ml/min/m^2^. In a study by Kai et al., the incidence of the no-reflow phenomenon was 11%, and this study also showed the eGFR value to be an independent predictor for the occurrence of the slow-flow/no-reflow phenomenon as well as for ST-segment resolution (STR). In this study, the eGFR was determined at hospital admission, and all patients were included in the study, irrespective of their eGFR values, while the prognostic impact of individual levels of kidney dysfunction was not analyzed. In this paper, the patients with the slow-flow/no-reflow phenomenon also had a lower average value of the baseline eGFR, which was identical to our findings [2]. In a paper by Celik et al., including 80 patients with STEMI, it was shown that the value eGFR <60 ml/min/m^2^ was an independent predictor of poor myocardial perfusion after primary PCI [5], which is a higher level of kidney dysfunction as compared to the findings in our study. The clinical findings from the study by Jinnouchi et al. are similar to those found in the aforementioned studies but obtained through a different approach. Namely, in this study, it was found that, in patients with acute myocardial infarction, preserved kidney function, i.e., a higher eGFR, was an independent predictor of transient no-reflow, i.e., of the normalization of blood flow through the IRA [8]. The results of all the above-mentioned studies, as well as the results of our study, indicate the great significance of preserved kidney function for successful primary PCI.

As the slow-reflow/no-reflow phenomenon signifies unsuccessful reperfusion in patients with STEMI, consequently, this phenomenon correlates with greater myocardial damage, remodeling of the left ventricle, the occurrence of different complications, and the lethal outcome, both in short-term and long-term follow-up [1, 3, 5, 8, 9, 11, 20–24]. Ndreppa et al. showed the no-reflow phenomenon as an independent predictor of five-year mortality upon STEMI [25]. On the other hand, there are studies showing that, even though mortality in patients with the no-reflow phenomenon has proven to be significantly higher in short-term and long-term follow-up, the no-reflow phenomenon was an independent predictor in these studies only during the 30-day follow-up [6]. In our study, we have shown this negative prognostic impact to persist during an eight-year follow-up, although we registered the highest mortality during the first 30 days of follow-up.

As already mentioned, the pathophysiology of the slow-flow/no-reflow phenomenon is complex and insufficiently understood [3, 8, 10]. Some of the causes of the slow-flow/no-reflow phenomenon stated in the literature include tissue swelling, injury to the endothelium, capillaries occluded by microthrombi and neutrophils, the build-up of free radicals, complement activation, etc. [2–5, 22]. The possible “individual tendency” towards developing this phenomenon should also be taken into consideration [1, 3]. It is known that patients with chronic kidney disease have chronic inflammation as well as abnormal thrombocyte activation, progressive atherosclerosis, and other numerous disorders that can be linked to the slow-flow/no-reflow phenomenon [3, 4, 26]. The occurrence of oxidative stress is also very important in patients with kidney dysfunction [2, 4]. It has also been shown that, in patients with chronic kidney disease, atherosclerotic plaques have a higher lipid index as compared to patients with preserved kidney function [2]. A larger lipid index in atherosclerotic plaque rupture may lead to distal embolization and blockage in the microcirculation [2, 20]. In decreasing kidney function, the elevation of the levels of the von Willebrand factor and of the C-reactive protein has been registered, as well as increased expression of the adhesive molecules on the endothelium [3, 11]. It is believed that all of these mechanisms already exist even in the mildest forms of kidney dysfunction, which may be the explanation for the frequent occurrence of the slow-flow/no-reflow phenomenon as soon as there is even a mild decrease in eGFR while the value of serum creatinine remains normal [5]. Also, it has been demonstrated that decreasing kidney function is linked to the decreased vasodilatory capacity of blood vessels in patients with obstructive coronary artery disease [2]. Of course, it must be kept in mind that decreased kidney function is often present in patients with diabetes mellitus and/or hypertension, which may also influence the occurrence of the said dysfunctions at the level of the microcirculation as well as the occurrence of diffuse atherosclerotic abnormalities [26]. In our study, a higher percentage of patients with diabetes mellitus were registered in the group with the slow-flow/no-reflow phenomenon. However, in multivariable logistic regression analysis, decreased kidney function remained an independent predictor of the slow-flow/no-reflow phenomenon, even when variables that were different in the preliminary analysis (including diabetes mellitus) were included in the model.

Clinical significance of the study: Our findings may add to the existing knowledge on the link between renal function and coronary disease, i.e., its impact on the occurrence of different complications and the outcome in patients with STEMI. Even STEMI patients with the mildest kidney function decline may have a higher risk of a poorer TIMI flow grade upon the opening of the IRA, which will have a strong negative impact on their short- and long-term prognosis.

### 4.1. Study Limitations

The study is observational, but it is controlled, prospective, and has included consecutive patients with no missing data, limiting possible selection bias. Angiographic assessments for postprocedural flow through the IRA, such as myocardial blush and TIMI frame count, were not used; therefore, the rate of slow-flow/no-reflow phenomena may have been under-reported. Kidney function at hospital admission can be a chronic condition or an acute decline of kidney function. Since, in the present study, the average time elapsing from the onset of symptoms to hospital admission was around three hours, and since blood for determining creatinine levels was taken immediately upon admission (and prior to pPCI and the use of the contrast agent), it is believed by the authors of the present study that the values of eGFR in the patients included in the study were predominately an indicator of a chronic state rather than of a temporary worsening. Also, patients with cardiogenic shock at admission were excluded from the study. The eGFR value of <90 ml/min/m^2^ was considered a reduced baseline eGFR; however, in some patients, this may solely be a reflection of age-related physiological decline. Kidney function was assessed with the use of the MDRD equation, which also has its limitations [17, 27, 28]. The rates of urinary albumin and protein excretion were not measured, and these are factors that may influence the independent impact of kidney function on the risk of the development of the slow-flow/no-reflow phenomenon [17, 26]. All patients included in our registry were treated with clopidogrel; there were no patients treated with more recently developed antiplatelet drugs (prasugrel and/or ticagrelor), and pPCI was predominantly performed using bare metal stents. Ticagrelor, prasugrel, and/or the new generation of drug-eluting stents or biodegradable polymers were not available for routine administration to patients at the time of their enrollment in the register, which may have influenced the prognosis of the analyzed patients. The study was not designed to evaluate whether changing pharmacological treatment would have had an impact on the slow-flow/no-reflow phenomenon and long-term outcome in the analyzed patients.

## 5. Conclusion

Reduced kidney function at admission was an independent predictor for the occurrence of the slow-flow/no-reflow phenomenon in patients with STEMI treated with primary PCI, and its prognostic impact started as soon as there was even the mildest decrease in eGFR (below 90 ml/min/m^2^) and increased with its further decline. The slow-flow/no-reflow phenomenon was a strong independent predictor for mortality in the short- and long-term follow-up of the analyzed patients.

## Figures and Tables

**Table 1:** Baseline characteristics, kidney function, and in-hospital mortality according to the postprocedural TIMI flow grade.

Characteristics	TIMI = 3	Slow-flow/no-reflow	*p* value
	*N* = 2969	*N* = 146	
Age, years med (IQR)	60 (51, 69)	65 (59, 72)	<0.001
Female, *n* (%)	819 (27.5)	57 (39)	0.006
Previous MI, *n* (%)	296 (10)	30 (20.5)	<0.001
Previous AP, *n* (%)	216 (7.3)	21 (14.4)	0.002
Previous PCI, *n* (%)	78 (2.6)	6 (4.1)	0.281
Diabetes, *n* (%)	570 (19.2)	39 (26.7)	0.026
Hypertension, *n* (%)	1990 (67)	99 (67.8)	0.958
HLP, *n* (%)	1918 (61.3)	70 (47.9)	0.001
Smoking, *n* (%)	1605 (54)	55 (37)	<0.001
Pain duration, hours med (IQR)	2.5 (1.5, 4.5)	3 (1.5, 5)	0.036
Atrial fibrillation at admission, *n* (%)	185 (6.2)	30 (20.5)	<0.001
Complete AV block at admission, *n* (%)	130 (4.4)	13 (8.9)	<0.001
HF at admission, *n* (%)	331 (11.2)	59 (44.6)	<0.001
Systolic BP (mmHg) at admission, med (IQR)	140 (120, 150)	130 (110, 150)	0.024
HR at admission, med (IQR)	80 (70, 90)	85 (70, 110)	<0.001
Anterior infarction, *n* (%)	1180 (39.7)	77 (52.7)	0.007
Multivessel disease, *n* (%)	1658 (55.1)	104 (71.2)	<0.001
Culprit vessel			
LAD, *n* (%)	1181 (39.8)	54 (36.9)	
Cx, *n* (%)	628 (21.2)	43 (29.4)	
RCA, *n* (%)	984 (33.1)	39 (26.7)	
LM, *n* (%)	176 (5.9)	10 (6.8)	
Stent implantation, *n* (%)	2829 (95.2)	132 (90.4)	0.045
Stent length, med (IQR)	23 (18, 26)	24 (23, 28)	0.010
Stent diameter, med (IQR)	3 (3, 3.5)	3.1 (3, 3.5)	0.897
Preprocedural flow TIMI 0, *n* (%)	2017 (67.9)	130 (89)	<0.001
IIb/IIIa receptor blockers, *n* (%)	1064 (34.2)	86 (58.9)	<0.001
CK, med (IQR)	1869 (997, 3467)	2560 (778, 4561)	0.079
Troponin I (*μ*g/L) med (IQR)	30 (19.6, 88)	34.6 (17.1, 110)	0.105
Hemoglobin g/L, med (IQR)	142 (131, 153)	136 (130, 147)	0.075
Creatinine at admission *μ*mol/L, med (IQR)	83 (70, 97)	88 (71, 100)	<0.001
eGFR ml/min/m^2^ med (IQR)	92.4 (70.4, 114)	77.5 (59.7, 97.8)	<0.001
eGFR ≥90 ml/min/m^2^, *n* (%)	1498 (51.7)	16 (11.1)	<0.001
eGFR 60–89 ml/min/m^2^, *n* (%)	1031 (34.7)	83 (56.8)	<0.001
eGFR 45–69 ml/min/m^2^, *n* (%)	283 (9.8)	25 (17.7)	<0.001
eGFR 30–44 ml/min/m^2^, *n* (%)	115 (4)	13 (8.9)	<0.001
eGFR 15–29 ml/min/m^2^, *n* (%)	42 (1.5)	8 (5.5)	<0.001
LVEF (%), med (IQR)	50 (40.55)	40 (50.30)	<0.001

Med = median; IQR = interquartile range; AP = angina pectoris; AV = atrioventricular; HLP = hyperlipidemia; MI = myocardial infarction; HF = heart failure; BP = arterial blood pressure; HR = heart rate; LAD = left anterior descending coronary artery; Cx = circumflex coronary artery; RCA = right coronary artery; LM = left-main coronary artery; CK = creatinine kinase; LVEF = left ventricular ejection fraction; eGFR = estimated glomerular filtration rate.

**Table 2:** Therapy during hospitalization and at discharge from hospital.

	TIMI = 3	Slow-flow/no-reflow	*p* value
*In-hospital*	*N* *=* *2969*	*N* *=* *146*	
Aspirin, *n* (%)	2969 (100)	145 (99.99)	0.785
Clopidogrel, *n* (%)	2967 (99.99)	142 (97)	0.870
Heparin, *n* (%)	2554 (86.7)	146 (100)	0.009
Beta blockers, *n* (%)	2583 (87)	109 (75)	0.504
ACE inhibitors, *n* (%)	2211 (74.5)	92 (63.1)	0.001
Statin, *n* (%)	2227 (74.7)	107 (73.3)	0.145
Diuretics, *n* (%)	292 (13.2)	66 (45.5)	<0.001

*At discharge*	*N* *=* *2882*^*∗*^	*N* *=* *102*^*∗*^	
Aspirin, *n* (%)	2882 (100)	102 (100)	0.955
Clopidogrel, *n* (%)	2850 (98.89)	92 (90.25)	0.552
Beta blockers, *n* (%)	2496 (86.7)	65 (63.7)	<0.001
ACE inhibitors, *n* (%)	2124 (73.7)	48 (47.1)	<0.001
Statins, *n* (%)	2880 (99.9)	100 (98.1)	0.985
Diuretic, *n* (%)	205 (6.9)	46 (45.5)	<0.001

^*∗*^number of discharged patients.

**Table 3:** Univariate and multivariate logistic regression analysis for the predictors of the slow-flow/no-reflow phenomenon.

Variable	Univariate analysis	*p* value	Multivariate analysis	*p* value
	OR 95% CI		OR 95% CI	
Age, (years)	1.03 (1.02–1.05)	0.001		
Killip class >1 at admission	5.43 (3.68–7.45)	<0.001	3.34 (2.28–5.41)	<0.001
Preprocedural flow TIMI 0	3.09 (1.82–4.59)	<0.001	3.17 (1.91–5.75)	<0.001
Atrial fibrillation at admission	2.89 (2.53–5.97)	<0.001	1.91 (1.12–3.22)	0.016
Multivessel disease	2.57 (1.83–3.59)	<0.001	1.91 (1.18–3.28)	0.001
Complete AV block at admission	2.49 (1.48–4.02)	0.001		
Previous MI	2.32 (1.51–3.53)	0.001		
Female sex	1.94 (0.89–5.31)	0.004		
Diabetes	1.52 (1.04–2.23)	0.026		
eGFR <90 ml/min/m^2^	3.65 (2.83–4.82)	<0.001	2.91 (1.25–3.95)	<0.001
eGFR 60–89 ml/min/m^2^	2.06 (1.36–3.19)	0.001	1.94 (1.22–3.07)	0.005
eGFR 45–59 ml/min/m^2^	3.23 (1.93–5.29)	<0.001	2.55 (1.55–4.94)	<0.001
eGFR 30–44 ml/min/m^2^	4.38 (2.43–7.82)	<0.001	2.77 (1.43–5.25)	<0.001
eGFR 15–29 ml/min/m^2^	6.65 (3.91–15.29)	<0.001	5.84 (2.84–8.01)	<0.001

MI = myocardial infarction; AV = atrioventricular.

**Table 4:** Univariate and multivariate Cox regression analysis showing independent predictors for short- and long-term (one-year and eight-year) all-cause mortality.

Variable	Univariate analysis	*p* value	Multivariate analysis	*p* value
	OR (95% CI)		OR (95% CI)	
30-day all-cause mortality				
Age, (years)	1.07 (1.06–1.09)	<0.001	1.03 (1.02–1.05)	<0.001
EF (%)	0.84 (0.82–0.85)	<0.001	0.87 (0.85–0.89)	<0.001
Slow-flow/no-reflow	11.28 (7.95–16.03)	<0.001	3.28 (2.10–5.12)	<0.001
Killip class >1 at admission	9.62 (8.24–12.5)	<0.001	2.68 (1.65–5.12)	<0.001
New-onset AF	5.91 (4.14–8.45)	<0.001	1.52 (1.10–2.34)	0.050
Diabetes	2.14 (1.72–3.19)	<0.001		
eGFR <90 ml/min/m^2^	5.53 (3.15–8.71)	<0.001	1.75 (1.23–3.19)	0.025
Complete AV block at admission	3.60 (2.90–4.15)	<0.001		
Previous MI	2.29 (1.34–3.59)	<0.001		
One-year all-cause mortality				
Age, (years)	1.07 (1.06–1.08)	<0.001	1.01 (1.03–1.05)	<0.001
EF (%)	0.87 (0.86–0.88)	<0.001	0.98 (0.89–0.92)	<0.001
Slow-flow/no-reflow	8.24 (5.72–11.92)	<0.001	2.21 (1.56–3.13)	<0.001
Killip class >1 at admission	8.21 (6.19–11.32)	<0.001	2.20 (1.56–3.08)	<0.001
eGFR <90 ml/min/m^2^	4.40 (3.08–6.44)	<0.001	1.61 (1.03–3.37)	0.034
New-onset AF	4.38 (3.18–6.87)	<0.001		
Complete AV block at admission	3.19 (2.33–4.39)	<0.001		
Diabetes	2.18 (1.65–2.94)	<0.001		
Previous MI	2.07 (1.45–2.94)	<0.001		
Eight-year all-cause mortality				
Age, (years)	1.07 (1.06–1.08)	<0.001	1.04 (1.02–1.05)	<0.001
EF (%)	0.92 (0.91–0.94)	<0.001	0.91 (0.90–0.92)	<0.001
Slow-flow/no-reflow	8.24 (5.69–10.32)	<0.001	2.09 (1.49–2.89)	<0.001
Killip class >1 at admission	7.81 (3.79–10.87)	<0.001	1.74 (1.32–2.41)	<0.001
eGFR <90 ml/min/m^2^	4.45 (3.13–5.99)	<0.001	1.47 (1.07–2.02)	0.047
New onset AF	3.88 (2.87–5.3)	<0.001		
Complete AV block at admission	2.52 (2.01–6.01)	<0.001		
Diabetes	2.08 (1.52–2.72)	<0.001		
Previous MI	2.00 (1.41–2.84)	<0.001		

EF = left ventricular ejection fraction; AF = atrial fibrillation; MI = myocardial infarction; AV = atrioventricular; eGFR = estimated glomerular filtration rate.

## Data Availability

The data are available upon request to the corresponding author.
